# Cognitive function and cannabis use in first episodes of psychosis

**DOI:** 10.1192/j.eurpsy.2025.1688

**Published:** 2025-08-26

**Authors:** P. Fuentes-Pérez, P. Cordero Andrés, C. Ovejas-Catalán, R. Pérez Iglesias, P. Suárez Pinilla, C. Flor Gómez, D. De la Sierra Biddle, M. L. Ramírez Bonilla, J. Vázquez-Bourgon

**Affiliations:** 1 Valdecilla Biomedical Research Institute - IDIVAL; 2 Psychiatry Department, University Hospital Marqués de Valdecilla; 3 University of Cantabria, Santander, Spain

## Abstract

**Introduction:**

The association between cannabis use and cognitive functioning in individuals experiencing a first episode of psychosis (FEP) is inconsistent, with some studies reporting deterioration (Bogaty et al. J Psychiatr Res 2018; 99:22-32), others indicating improved performance (Rodríguez-Sánchez et al. Schizophr Res 2010; 124:142-151), and some finding no difference.

**Objectives:**

This study aims to evaluate the effect of cannabis use on cognitive functioning among individuals diagnosed with FEP.

**Methods:**

A cross-sectional study was conducted on FEP patients enrolled in the ITPCan Program (Santander, Spain) between January 2020 and July 2024. A total of 207 participants (57 cannabis users and 145 non-users) with a FEP diagnosis were included. A descriptive-univariate analysis was performed on clinical, analytical, and cognitive variables. A Student’s t-test compared baseline cognitive performance between cannabis users and non-users, while a multivariate general linear model (GLM) assessed differences related to cannabis consumption, adjusting for sex, age, and educational level. Statistical analyses were performed using SPSS.

**Results:**

Out of the 207 FEP participants, 53.6% were women, with an average age of 36.8 years. Cannabis users comprised 28.1% of the group, and present at baseline a lower mean age at intake (27 years; p < 0.001). Cannabis users exhibited significantly higher scores in mania and positive psychotic symptoms. Cognitive assessments, completed by 148 patients (39 users and 108 non-users), revealed that cannabis users performed better than non-users on “processing speed” task; however, after adjusting for sex, age, and educational level, these differences were attributed to educational level and sex.

**Conclusions:**

In consonance with some previous studies (Sánchez-Gutiérrez et al. Eur Psychiatry 2020; 63(1)), cannabis use does not appear to be a determining factor in cognitive performance in early psychosis.

**Disclosure of Interest:**

None Declared

